# Physics-informed structural diagnostics of model–data agreement beyond scalar metrics

**DOI:** 10.1038/s41598-026-49445-8

**Published:** 2026-04-22

**Authors:** Hedayat Haddadi, Adam Kloskowski, Piotr Mironowicz

**Affiliations:** 1 Department of Physical Chemistry, Faculty of Chemistry, Gdańsk University of Technology, 80–233 Gdańsk, Poland; 2 Department of Algorithms and Systems Modelling, Faculty of Electronics, Telecommunications and Informatics, Gdańsk University of Technology, 80–233 Gdańsk, Poland

**Keywords:** Mathematics and computing, Physics

## Abstract

Physics-informed machine-learning models increasingly incorporate physical laws and constraints to improve data efficiency and predictive robustness; yet their validation remains dominated by pooled scalar accuracy metrics that are largely insensitive to violations of the underlying governing relationships. Here we introduce a physics-informed validation framework, the Agreement–Entropy Map (AEM), which diagnoses model–data agreement by distinguishing structural incompatibility from conditional stochastic dispersion, rather than by defining a scalar metric or additive error decomposition. Conditioned on a physically motivated linearization of the governing relation and evaluated on matched comparison domains, AEM combines regression geometry with an information-theoretic dispersion measure based on a Gaussian plug-in entropy of residuals, without requiring distributional modeling or inferential assumptions. The framework applies uniformly to experiment–experiment and model–experiment comparisons and is agnostic to model class, architecture, and training procedure. Using thermodynamic systems as a canonical physics-governed testbed, we show that AEM reveals structural bias, variance-driven artefacts, and ensemble effects that remain undetected by conventional scalar validation metrics. By identifying when stochastic interpretation is admissible under a shared physical structure, AEM provides a general and interpretable validation principle for physics-informed machine learning, particularly in regimes involving limited, heterogeneous, or damaged data.

## Introduction

Recent advances in physics-informed machine learning have shown that embedding physical laws, symmetries, and governing equations into predictive models can substantially improve data efficiency, generalization, stability, and extrapolation across scientific and engineering domains^1–6^. These developments reflect a broader shift away from purely data-driven regression toward learning frameworks in which prior physical knowledge enters as an explicit structural constraint. As a result, physics-informed neural networks, neural operators, and related architectures are increasingly deployed in settings where predictive robustness and physical consistency are essential.

Despite this progress in model construction, the problem of *validation* has received comparatively less systematic attention. In practice, physics-informed and conventional machine-learning models alike continue to be assessed primarily using pooled scalar accuracy metrics such as RMSE, MAE, MAPE, SMAPE, or the coefficient of determination. A growing body of work has demonstrated that such metrics are mathematically and physically insufficient for evaluating fidelity when models are expected to respect known governing relationships or conservation laws^7–10^. By collapsing all discrepancies into a single aggregate score, scalar metrics are insensitive to whether disagreement arises from violations of physical structure or from stochastic variability, and they offer little diagnostic power for understanding model behavior outside the training domain^11^.

This limitation becomes especially acute in regimes involving limited, heterogeneous, or partially corrupted data, which are common in scientific measurement and engineering practice. In such settings, models may achieve apparently competitive accuracy while exhibiting systematic structural deviations from the governing physical law, leading to misleading assessments of robustness and trustworthiness. Addressing this challenge requires validation procedures that operate *relative to the implied physical relationship*, rather than treating prediction error as an undifferentiated residual.

A broad class of physics-informed models admits representations in which the governing relationship becomes linear, or approximately linear, after an appropriate physically motivated transformation. Canonical examples include van’t Hoff, Arrhenius, and Gibbs–Helmholtz relations in thermodynamics, as well as linearized regimes encountered in kinetics, transport, and other physics-governed systems. When two predictive mechanisms or data sources are expected to obey the same linearizable law, meaningful validation must therefore assess not only residual magnitude, but also whether they imply the same functional mapping over a shared comparison domain. Classical linear-model theory provides tools to formalize this distinction: restricted–unrestricted regression comparisons^12,13^ quantify agreement between functional relationships, while information-theoretic measures of residual disorder^14,15^ characterize stochastic dispersion conditional on a specified structure. To date, however, these concepts have not been unified into a general, physics-informed validation framework for machine-learning models.

Here we introduce the *Agreement–Entropy Map* (AEM), a physics-informed diagnostic framework for evaluating model–data agreement under known physical constraints. Conditioned on a physically motivated linearization and evaluated on matched comparison domains, AEM distinguishes violations of the governing functional relationship from stochastic variability that is meaningful only when such a shared structure is admissible, rather than defining a scalar metric or additive error decomposition. Structural incompatibility is identified through regression-based comparison of the implied functional mappings, while residual dispersion is characterized, conditional on structural admissibility, using a Gaussian plug-in entropy measure. The framework is agnostic to model class, architecture, and training procedure, and applies uniformly to model–model, model–experiment, and experiment–experiment comparisons. Using thermodynamic systems as a canonical physics-governed testbed, we show that AEM exposes structural bias, variance-driven artefacts, and ensemble effects that remain invisible to conventional scalar validation metrics. By identifying when stochastic interpretation is admissible under a shared physical structure, AEM provides a general and interpretable validation principle for physics-informed machine learning and data-driven scientific discovery.

The AEM is complementary to several existing strategies used in physics-informed learning. Many frameworks incorporate governing equations or conservation laws directly into model construction or training, for example by penalizing equation residuals at collocation points or by enforcing analytic constraints in the loss function or architecture^16,17^. Related operator-learning approaches instead focus on learning the input–output operators induced by physical systems^18^. In contrast, AEM addresses the distinct problem of validation: it evaluates whether two data-generating mechanisms imply compatible effective mappings on a shared domain and how their residual dispersion changes when the sources are combined. The framework therefore provides a structural–stochastic diagnostic of agreement that is complementary to equation-residual or constraint-based consistency checks rather than replacing them.

## Physics-informed validation framework

Such representations arise not only in thermodynamics (e.g., van’t Hoff, Arrhenius, and Gibbs–Helmholtz forms), but also in linearized rate and transport laws in continuum mechanics, log–linear scaling relations in biological allometry and population dynamics, stress–strain relations in solid mechanics, exponential-response laws in quantitative psychophysics, and log–linear models of molecular abundance in omics data.

When two predictive mechanisms or data sources are expected to obey the same underlying physical law, meaningful validation must therefore assess not only the magnitude of residuals, but also whether both sources imply the *same functional mapping* over a shared domain. The AEM formalizes this requirement by diagnosing structural incompatibility in the governing relationship and characterizing residual dispersion conditional on structural admissibility, rather than by defining a scalar metric or additive decomposition of error.

### Linearizable mappings and restricted–unrestricted regression geometry

We consider settings in which a physically or mechanistically motivated transformation yields an approximately linear relationship of the form^13^

1 $$\begin{aligned} y = a + b x + \varepsilon , \end{aligned}$$

where (*a*, *b*) denote the parameters of the linearized governing relationship and $\varepsilon$ is a zero-mean error term with finite variance. Under this representation, two datasets or predictive models describing the same mechanistic system are expected to yield compatible parameter estimates when evaluated over a common range of the independent variable. Restricting comparisons to this shared or overlap domain is therefore essential to ensure that observed discrepancies reflect genuine structural or stochastic differences rather than artefacts of unequal sampling support.

To assess agreement under this condition, AEM contrasts two least-squares regressions constructed on the overlap domain: (i) an *unrestricted* fit in which each source is fitted independently, yielding a combined residual sum of squares $\textrm{SSR}_u$, and (ii) a *restricted* pooled fit enforcing a single shared functional mapping, yielding $\textrm{SSR}_r$. The ratio $\textrm{SSR}_r / \textrm{SSR}_u$ quantifies the geometric penalty incurred by enforcing structural compatibility between sources and constitutes the fundamental restricted–unrestricted comparison underlying the AEM diagnostics.

### Structural deviation

In classical analysis of covariance, differences between regression relationships are assessed using the Chow statistic^12^,

$$F_{\textrm{Chow}} = \frac{(\textrm{SSR}_r - \textrm{SSR}_u)/k}{\textrm{SSR}_u/(n - 2k)} ,$$

which is derived under Gaussian and homoscedastic assumptions and interpreted within a hypothesis-testing framework. AEM does not adopt this inferential perspective and instead retains only the algebraic and geometric content of the restricted–unrestricted comparison.

Structural deviation is defined as the logarithmic ratio

2 $$\begin{aligned} S_{\textrm{struct}} = \frac{1}{2} \ln \!\left( \frac{\textrm{SSR}_r}{\textrm{SSR}_u} \right) , \end{aligned}$$

a dimensionless, nonnegative quantity that vanishes if and only if the restricted and unrestricted fits yield identical residual dispersion. This quantity measures the geometric penalty incurred by enforcing a shared functional mapping and is invariant to rescaling of the response variable. The logarithmic form places structural deviation on an information scale, facilitating direct comparison with the stochastic diagnostic introduced below, while not implying additivity or componentwise decomposition of error. Although $S_{\textrm{struct}}$ is derived from regression geometry, it aggregates deviations in both slope and intercept into a single diagnostic quantity. In many physical systems these parameters carry different mechanistic interpretations: intercept differences may arise from calibration offsets or reference-state conventions, whereas slope differences may reflect changes in the underlying physical relationship. Accordingly, $S_{\textrm{struct}}$ should be interpreted as a compact summary of structural incompatibility rather than a mechanistic decomposition. In applications where such distinctions are physically important, slope- or intercept-specific analyses may be considered alongside the AEM diagnostics.

### Stochastic deviation

Residual variability may differ across experimental datasets and predictive models due to measurement noise, heterogeneity in data generation, or differences in model capacity. AEM characterizes such effects using an entropy-motivated functional based on the classical expression for the differential entropy of a Gaussian random variable^14,15^,

3 $$\begin{aligned} H(\mathscr {N}(0,\sigma ^2)) = \frac{1}{2} \ln (2\pi e \sigma ^2). \end{aligned}$$

Because this quantity attains the maximum entropy among all distributions with fixed variance $\sigma ^2$, it provides a principled variance–entropy correspondence that depends only on second moments and does not require Gaussian residuals.

Let $\widehat{\sigma }_{\textrm{ref}}^2$ denote the residual variance of a reference source and $\widehat{\sigma }_{\textrm{pool}}^2$ the pooled residual variance obtained after combination with a comparator. The stochastic deviation is defined as

4 $$\begin{aligned} \Delta H = \frac{1}{2} \ln \!\left( \frac{\widehat{\sigma }^{2}_{\textrm{pool}}}{\widehat{\sigma }^{2}_{\textrm{ref}}} \right) , \end{aligned}$$

which quantifies the relative change in residual dispersion induced by pooling. Positive values correspond to dispersion inflation, whereas negative values indicate reduced residual disorder, conditional on structural compatibility. The entropy terminology here refers to a Gaussian plug-in variance functional rather than to an estimator of the full differential entropy of the residual distribution. Consequently, $\Delta H$ depends only on the second moment of the residuals and can equivalently be interpreted as a logarithmic variance ratio expressed on an information-theoretic scale. The stochastic coordinate therefore captures variance-mediated disagreement between sources but does not resolve higher-order distributional features such as skewness, heavy tails, or multimodality. In applications where such effects are physically meaningful, complementary diagnostics may be required in addition to the AEM coordinates.

### AEM coordinates and joint deviation

For a model or dataset *M* evaluated against a reference *R*, the AEM coordinates are defined as

$$x_M = S_{\textrm{struct}}(M), \qquad y_M = \Delta H(M).$$

Both coordinates are dimensionless and logarithmic, representing distinct but non-additive diagnostics of structural compatibility and stochastic dispersion. Because the stochastic coordinate measures a variance ratio relative to a designated reference, $\Delta H$ may in some cases take negative values. Such outcomes occur when pooling the comparator with the reference reduces the overall residual dispersion. This situation does not necessarily imply improved physical agreement; it may arise from variance contraction, smoothing effects, or partial cancellation of systematic biases. For this reason, negative stochastic deviation should be interpreted jointly with the structural coordinate rather than as an isolated indicator of model fidelity. For compact visualization and summary purposes, they may be combined into a scalar diagnostic

5 $$\begin{aligned} d_{\textrm{AEM}}(M) = \sqrt{x_M^2 + y_M^2}. \end{aligned}$$

Any such scalarization represents a pragmatic aggregation rather than a representation-invariant characterization. Distinct combinations of structural and stochastic deviation may therefore yield identical scalar values. Accordingly, $d_{\textrm{AEM}}$ is intended solely as a practical summary quantity and does not replace the two-dimensional AEM representation, which retains the information necessary to distinguish the underlying sources of disagreement.

### Scope and assumptions

The AEM framework applies to settings in which a physically or mechanistically motivated transformation yields a linear or approximately linear representation of the governing relation over a shared comparison domain. Under these conditions, the restricted–unrestricted regression geometry provides a natural mechanism for diagnosing whether two sources imply the same effective mapping and for characterizing changes in residual dispersion.

The present formulation therefore requires that (i) a physically meaningful linear or approximately linear representation be available, (ii) comparisons be conducted on a matched domain of the explanatory variable, and (iii) residuals be expressed in common physical units with finite variance. No assumptions of Gaussianity, homoscedasticity, or statistical inference are invoked.

These conditions define the practical scope of the framework. Systems governed by strongly nonlinear, multivariate, operator-valued, or history-dependent relations may not admit a stable linearizable representation on a shared domain, in which case the present construction may be less appropriate or require extension. Similarly, very sparse overlap domains or weak signal-to-noise ratios may limit the interpretability of restricted–unrestricted comparisons. The examples considered here therefore correspond to settings in which a physically motivated linearization provides a meaningful diagnostic reference.

## Limitations of scalar accuracy metrics

**Fig. 1:**
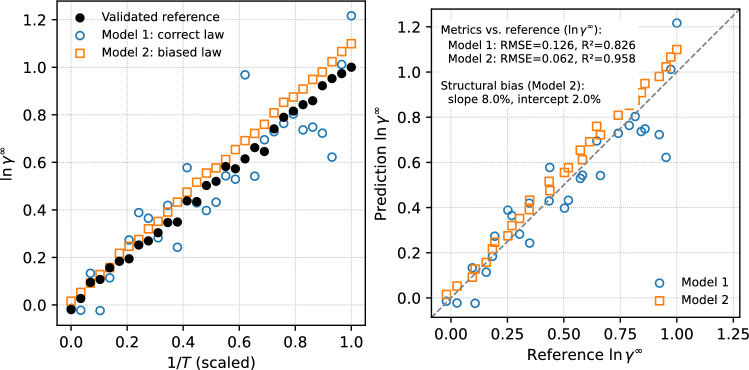
Structural blind spot of conventional validation metrics. A reference linearized governing relationship (black) is compared with two predictive models: one preserving the physically correct slope (Model 1, blue) and one exhibiting a biased slope but reduced pointwise variability (Model 2, orange). Although Model 2 visibly violates the governing relationship, it achieves superior RMSE and $R^{2}$ values. Because pooled scalar metrics are insensitive to structural incompatibility, they cannot distinguish faithful representations of a physical mapping from biased but numerically tighter fits.

Scalar accuracy metrics such as RMSE, MAE, and the coefficient of determination are designed to summarize the magnitude of pooled residuals and are intentionally agnostic to the functional form of the underlying relationship. As a consequence, they provide no mechanism for assessing whether a predictive model preserves the *structure* implied by physical laws, symmetries, or constraints. A substantial body of work has shown that RMSE- and MAE-based rankings can be misleading in the presence of heteroscedastic, skewed, or heavy-tailed errors^7^, that $R^{2}$ may yield inconsistent or erroneous conclusions even in simple regression settings^9,10^, and that magnitude-only metrics can fail to detect physically implausible behavior^11^.

Figure 1 illustrates a failure mode that is particularly consequential for physics-informed machine learning. A model that violates the governing functional relationship may nevertheless achieve lower RMSE or higher $R^{2}$ than a structurally faithful model if its residual variance is reduced. In such cases, scalar metrics implicitly trade physical fidelity for numerical tightness, conflating structural deviation with stochastic dispersion. This confounding is exacerbated in regimes involving limited, heterogeneous, or partially corrupted data, where variance reduction may arise from over-regularization, biased training distributions, or model-capacity effects rather than improved adherence to the governing law.

These considerations expose a structural limitation of scalar accuracy metrics for validating physics-informed predictive models. By collapsing distinct sources of disagreement into a single numerical summary, they cannot provide mechanistic assurance of model validity or physical consistency. This motivates the use of diagnostic frameworks, such as the AEM, that distinguish structural incompatibility from stochastic dispersion and evaluate model–data agreement relative to the implied physical relationship, rather than treating them as additive components of a single error term.

## Methods

### Experimental datasets and preprocessing

Experimental data were obtained from the ILThermo database, a curated infrastructure that compiles critically evaluated thermodynamic measurements reported by independent laboratory sources^19,20^. Thermodynamic systems are used here as a canonical physics-governed testbed; however, the preprocessing and comparison procedures described below are generic and apply to any setting in which the governing relationship admits a linear or approximately linear representation. Thermodynamic activity-coefficient systems constitute a relatively favorable regime for illustrating the proposed diagnostics. The governing relationships are well established, physically motivated linearizations such as van’t Hoff or Gibbs–Helmholtz forms are canonical, and independent measurements are often available across comparable temperature ranges. These properties enable structural and stochastic deviations to be interpreted with minimal ambiguity. In more complex settings–such as systems with sparse overlap domains, weak signal-to-noise ratios, or competing linearizable representations–the interpretation of AEM coordinates may require additional care.

Activity-coefficient measurements were selected as the target property. For each chemical system, data were grouped by literature source. Because the objective is to assess inter-source coherence rather than absolute accuracy, only systems with at least two independent reports were retained, irrespective of whether those reports originated from distinct publications or from multiple datasets within a single study. All pairwise comparisons were restricted to the maximal common temperature domain shared by the sources, ensuring that structural and stochastic differences are not confounded by unequal sampling ranges. No interpolation, smoothing, variance weighting, or data augmentation was applied. Comparisons were performed only when at least three overlapping observations were available, ensuring that restricted–unrestricted regressions were well-defined.

The resulting curated collection of paired subsets constitutes the experimental basis for structural and stochastic deviation analysis under the AEM framework. For each retained pair, the structural deviation $S_{\textrm{struct}}$ and the stochastic deviation $\Delta H$ were computed to characterize agreement in the implied governing relationship and the associated residual dispersion on the matched comparison domain.

### Truth-anchored evaluation of predictive models

Truth-anchored model evaluation was performed using the benchmark dataset of Rittig *et al.*^21^, which provides experimentally measured reference values together with predictions from two contemporary machine-learning frameworks: (i) a graph neural network (GNN) and (ii) a matrix-completion model (MCM) inspired by Chen *et al.*^22^. Each model family comprises forty independently trained realizations together with their ensemble mean, yielding forty-one predictors per class.

Model predictions were aligned with experimental reference measurements at matching temperatures, and systems with fewer than three aligned observations were excluded to ensure that restricted–unrestricted regressions were well-defined. For each individual model realization, the structural deviation $S_{\textrm{struct}}$ and the stochastic deviation $\Delta H$ were computed relative to the experimental reference using the AEM definitions introduced in “Physics-informed validation framework” section. These quantities diagnose, respectively, incompatibility with the governing functional relationship and changes in residual dispersion relative to experimental truth, with stochastic dispersion interpreted conditional on the structural comparison.

For compact comparison, the two coordinates may be summarized through the joint diagnostic

$$d_{\textrm{AEM}} = \sqrt{S_{\textrm{struct}}^{2} + \Delta H^{2}},$$

with the understanding that identical scalar values may correspond to distinct combinations of structural and stochastic deviation. Because the benchmark contains multiple independent realizations within each model family, this setting enables characterization of both ensemble-level behavior and intra-family variability in physics-informed agreement with the experimental record.

### Computation of coherence metrics and model ranking

For each model realization and each valid system, the AEM coordinates were computed from the restricted–unrestricted residual sum-of-squares ratio and the anchored variance ratio,

$$S_{\textrm{struct}} = \tfrac{1}{2} \ln \!\left( \frac{\textrm{SSR}_{r}}{\textrm{SSR}_{u}}\right) , \qquad \Delta H = \tfrac{1}{2} \ln \!\left( \frac{\widehat{\sigma }^{2}_{R\cup M}}{\widehat{\sigma }^{2}_{R}} \right) ,$$

yielding the system-level joint diagnostic

$$d_{\textrm{AEM}} = \sqrt{S_{\textrm{struct}}^{2} + \Delta H^{2}}.$$

To summarize behavior across systems and enable comparative analysis, the following descriptive statistics were reported:

1. **Domain coverage**
  $$N_{\textrm{valid}} = \#\{ j : (S_{\textrm{struct},j}, \Delta H_j) \text { are finite} \},$$
  representing the number of systems for which a coherent model–reference comparison was well defined.
2. **Typical behavior** The medians of $S_{\textrm{struct}}$, $\Delta H$, and $d_{\textrm{AEM}}$, characterizing central tendencies in structural deviation, stochastic dispersion, and their joint diagnostic.
3. **Tail behavior** The $5^{\textrm{th}}$ and $95^{\textrm{th}}$ percentiles of $d_{\textrm{AEM}}$, capturing robustness and the prevalence of extreme joint deviations in the AEM coordinates.
4. **Coherence coverage** For a prescribed diagnostic radius $r^{*}$,
  $$\pi (r^{*}) = \Pr (d_{\textrm{AEM}} < r^{*}),$$
  representing the fraction of systems lying within a specified region of joint structural and stochastic agreement.

All predictors, including ensemble means, were evaluated using identical procedures to ensure comparability across model families. Model ordering based on these summaries is intended as a descriptive aid rather than an objective or representation-invariant ranking, as distinct combinations of structural and stochastic deviation may yield similar scalar summaries and the two-dimensional AEM coordinates remain the primary diagnostic output.

### Overlap-gated comparison and AEM construction

The AEM is constructed by comparing two data sources or predictive mechanisms only on their maximal shared domain of the transformed explanatory variable. For linearizable governing relations of the form

$$\ln K = a + b\,\frac{1}{T} + \varepsilon , \qquad x=\frac{1}{T}>0,$$

this overlap gating defines a comparison interval

$$\Omega = [x_{\min }, x_{\max }], \qquad x_{\min }=\max (\min x_1,\min x_2),\; x_{\max }=\min (\max x_1,\max x_2),$$

ensuring that subsequent diagnostics reflect structural and stochastic differences rather than mismatched sampling support.

On $\Omega$, structural agreement is assessed by contrasting restricted (pooled) and unrestricted (separate) least-squares regressions, while stochastic agreement is evaluated through entropy-based variance lifts computed from residuals. Depending on context, the stochastic coordinate may be defined in an anchored form relative to a designated reference or in a symmetric form treating both sources on equal footing. These constructions together yield the two-dimensional AEM representation.

A complete numerical illustration of the restricted–unrestricted regression geometry, overlap gating, anchored and symmetric stochastic coordinates, and the resulting AEM distances is provided in Section S3 of the Supporting Information.

### System-level aggregation

Model evaluation is conducted across many distinct systems, where a *system* denotes a specific physical interaction context characterized by its own linearized governing relationship and residual structure. System-level AEM diagnostics therefore constitute a heterogeneous distribution, rather than repeated observations of a single underlying process.

To summarize overall model behavior without allowing a small number of idiosyncratic or poorly behaved systems to dominate the assessment, aggregation is performed using robust descriptive statistics. In particular, the median of $d_{\textrm{AEM}}$ captures the characteristic structural–stochastic coherence of each model family while preserving the distributional spread of system-specific deviations as diagnostically informative.

### Simulation studies

To elucidate how distinct physical and statistical deviations populate the AEM plane, four controlled simulation scenarios were constructed: structural mismatch, variance imbalance, mixture residuals, and local nonlinearity. Synthetic datasets were generated from a common linear baseline relationship, with prescribed perturbations applied to the slope, intercept, or residual structure. For each scenario, AEM coordinates were computed across repeated realizations, and the resulting distributions were used to identify characteristic geometric signatures associated with each deviation type.

All computational workflows, scripts, and reproducible code are available in the public repository associated with this study.

## Results and discussion

### Complementarity of structural and stochastic diagnostics

**Fig. 2:**
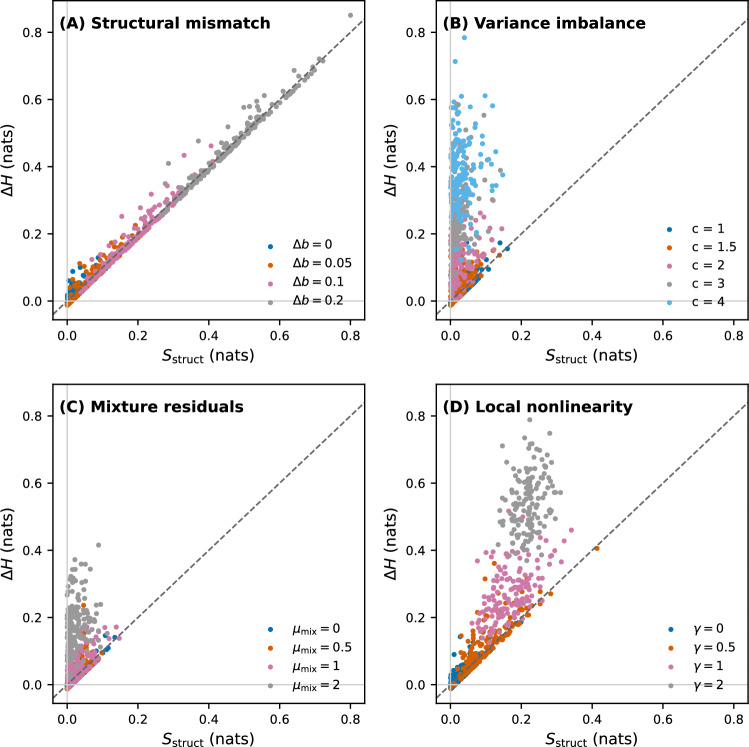
Complementarity of structural and stochastic diagnostics in the AEM. Simulated subset pairs illustrate four distinct mechanisms of disagreement: (**A**) structural mismatch between linear laws at fixed noise level; (**B**) variance imbalance under a shared linear law; (**C**) symmetric mixture residuals inducing marginal variance inflation; and (**D**) smooth quadratic curvature violating linearizability without altering intrinsic noise. The dashed line indicates $\Delta H = S_{\textrm{struct}}$.

Figure 2 illustrates the response of the two AEM coordinates employed in this work to four controlled mechanisms of disagreement. In each case, a single aspect of the data-generating process–structural form, residual variance, residual composition, or local nonlinearity–is perturbed while all other features are held fixed. This controlled design enables interpretation of $S_{\textrm{struct}}$ and $\Delta H$ in expectation, without confounding between independent sources of deviation.

The resulting patterns demonstrate that the selected coordinates capture complementary and non-redundant aspects of model–data disagreement. Pure structural mismatch produces horizontal displacement in the AEM plane with minimal change in $\Delta H$, whereas variance imbalance induces vertical displacement with negligible structural penalty. Mixture residuals generate modest but symmetric dispersion inflation, while local curvature produces a pronounced structural deviation despite unchanged intrinsic noise. These behaviors are consistent with the geometry of restricted–unrestricted regressions^13^ and with the variance–entropy correspondence implied by the Gaussian maximum-entropy functional^15^.

Importantly, the choice of $S_{\textrm{struct}}$ and $\Delta H$ is neither unique nor exhaustive. They constitute a minimal, physically interpretable pair of diagnostics appropriate for linearizable governing relationships and variance-mediated stochastic effects. Depending on the application, additional coordinates may be introduced to resolve other sources of disagreement–such as higher-order nonlinear structure, domain-dependent heteroscedasticity, temporal correlation, or distributional shape effects–leading to higher-dimensional extensions of the AEM. The present two-dimensional construction should therefore be understood as a foundational instance of a more general physics-informed diagnostic geometry, rather than as a mandatory or closed choice of coordinates.

#### Structural mismatch

When two subsets follow linear laws with different slopes but share identical noise levels, AEM points align closely with the one-to-one relation $\Delta H \approx S_{\textrm{struct}}$. In this regime, residual variance inflation arises entirely from the geometric penalty incurred by enforcing a shared linear mapping, rather than from any independent stochastic mechanism. Consequently, the entropy-based coordinate mirrors the structural deviation, consistent with the restricted–unrestricted sum-of-squares decomposition that underlies Chow’s equality-of-slopes analysis^12^. This diagonal alignment reflects an entropy reparameterization of the restricted–unrestricted geometry under a Gaussian plug-in construction, rather than the coexistence of distinct structural and stochastic effects.

#### Variance imbalance

If two subsets share the same underlying linear law but exhibit unequal residual variances, $S_{\textrm{struct}}$ remains small in expectation, whereas $\Delta H$ increases systematically as the variance ratio departs from unity. The resulting AEM cloud is therefore approximately vertical, with scatter in $S_{\textrm{struct}}$ arising primarily from finite-sample effects. The weak coupling between the two coordinates indicates that the structural diagnostic is largely insensitive to heteroscedasticity in this regime. Here the stochastic coordinate reflects variance-driven disagreement through its logarithmic dependence on residual dispersion.

#### Mixture residuals

When one subset exhibits symmetric mixture-distributed residuals while preserving the same underlying linear law, $S_{\textrm{struct}}$ again remains small in expectation, up to finite-sample variability. Because the stochastic coordinate is defined through a Gaussian plug-in entropy, it responds only to changes in the second moment of the residual distribution. Accordingly, $\Delta H$ increases due to mixture-induced variance inflation, while higher-order distributional shape effects are not resolved. The weak coupling between coordinates therefore indicates that variance-mediated stochastic incoherence is captured largely independently of structural alignment.

#### Local nonlinearity

Introducing smooth quadratic curvature within the overlap domain generates a structural perturbation that cannot be represented by a single linear mapping. Although the nominal linear coefficients are identical in both subsets, the curvature alters the effective least-squares representation and increases the restricted–unrestricted residual sum of squares. Residual variance is secondarily inflated under linear regression due to model misspecification rather than intrinsic noise heterogeneity. As a result, both $S_{\textrm{struct}}$ and $\Delta H$ increase, with their relative magnitudes depending on the strength of the curvature. Such mixed signatures cannot be resolved by classical Chow tests or by single-axis accuracy metrics, which are unable to disentangle nonlinear bias from variance inflation^13^.

Taken together, these scenarios demonstrate that the structural and stochastic axes of the AEM respond in complementary and diagnostically distinct ways to different mechanisms of disagreement. Pure structural violations align closely with the $\Delta H = S_{\textrm{struct}}$ relation, reflecting residual inflation driven by enforced model sharing rather than independent noise effects. Purely variance-driven perturbations produce predominantly vertical displacements, while mixture-induced variance inflation elevates $\Delta H$ with minimal structural response. Nonlinear distortions occupy an intermediate region of the AEM plane, where structural misspecification induces secondary stochastic inflation under linear regression. This complementarity underlies the interpretability of AEM and enables mechanistic insight into the origin of disagreement that is inaccessible to conventional scalar accuracy metrics.

### Truth-anchored coherence of ensemble predictions

**Fig. 3:**
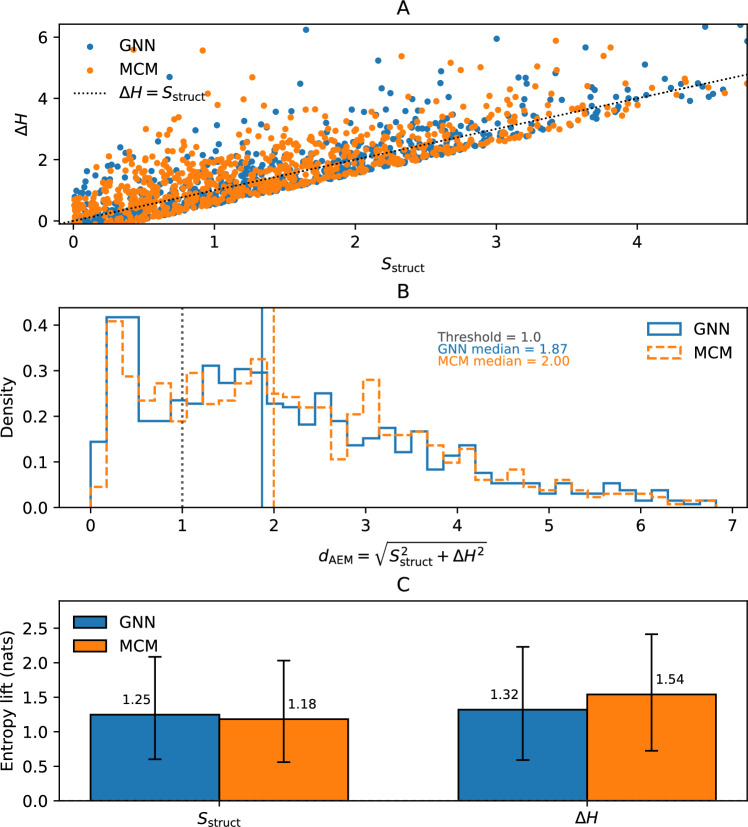
Truth-anchored AEM analysis of predictions from GNN and MCM models. (**A**) Structural–stochastic scatter for ensemble predictors, showing comparable structural deviation but distinct stochastic dispersion between families. (**B**) Distributions of the joint diagnostic $d_{\textrm{AEM}}$ for ensembles, illustrating substantial system-level heterogeneity. (**C**) Median values of the AEM coordinates, summarizing structural deviation and stochastic dispersion for each ensemble. The AEM coordinates diagnose geometric misalignment in the linearized mapping and variance-driven changes in residual dispersion, enabling system-resolved validation beyond pooled scalar accuracy metrics.

To quantify agreement between predictive models and experimental reference data, truth-anchored AEM coordinates were computed for all systems in the GNN and MCM model families, both for individual realizations and for the corresponding ensemble predictors (Table 1 and Fig. 3). The structural coordinate $S_{\textrm{struct}}$ reflects disagreement in the linearized governing relationship through restricted–unrestricted regression geometry^12,13^, whereas the stochastic coordinate $\Delta H$ quantifies changes in residual dispersion induced by pooling model and experimental data via a Gaussian plug-in entropy functional^14,15^.

At the ensemble level, the two model families exhibit broadly similar structural behavior. Median values of $S_{\textrm{struct}}=1.25$ for GNN and 1.18 for MCM indicate that both ensembles reproduce the linearized thermodynamic mapping with modest but systematic structural deviation. No ensemble-level comparisons yield negative structural deviation, implying that slope and intercept mismatches are present across many systems but remain consistent within each model family.

Despite similar structural behavior, the ensembles differ more substantially along the stochastic axis. The GNN ensemble exhibits a median entropy lift of $\Delta H=1.32$, whereas the MCM ensemble yields $\Delta H=1.54$; the fractions of systems with reduced dispersion upon pooling, $\Pr (\Delta H<0)$, are 0.09 and 0.08, respectively (Table 1). Thus, the two ensembles occupy distinct regions of the stochastic axis even when their structural deviations align closely.

When summarized through the joint diagnostic $d_{\textrm{AEM}}=\sqrt{S_{\textrm{struct}}^{2}+\Delta H^{2}}$, the median values are 1.87 for the GNN ensemble and 2.00 for the MCM ensemble. The corresponding fractions $\Pr (d_{\textrm{AEM}}<1)$ are 0.26 and 0.23, indicating that both ensembles contain similar proportions of highly coherent systems. For both families, the 5th and 95th percentiles of $d_{\textrm{AEM}}$ lie near 0.27–0.30 and 5.13–5.29, respectively, revealing a broad distribution in which a subset of systems agrees closely with experiment while another subset exhibits pronounced incoherence, despite comparable aggregate accuracy.

Comparison with individual base models clarifies how ensemble averaging redistributes systems across structural and stochastic modes of disagreement. For the GNN family, base models exhibit median values $S_{\textrm{struct}}=1.18$, $\Delta H=1.85$, and $d_{\textrm{AEM}}=2.27$, with $\Pr (d_{\textrm{AEM}}<1)=0.17$ and $\Pr (\Delta H<0)=0.05$. For the MCM family, the corresponding medians are $S_{\textrm{struct}}=1.20$, $\Delta H=1.87$, and $d_{\textrm{AEM}}=2.32$, with $\Pr (d_{\textrm{AEM}}<1)=0.16$ and $\Pr (\Delta H<0)=0.05$. In both cases, ensemble averaging substantially reduces stochastic deviation and joint dispersion, increases the fraction of highly coherent systems, and leaves the upper tail of $d_{\textrm{AEM}}$ largely unchanged. From the AEM perspective, ensemble averaging therefore does not yield a uniformly superior predictor, but instead reshapes the distribution of system-level coherence across structural and stochastic dimensions.

In this role, the truth-anchored AEM framework complements thermodynamics-consistent machine-learning approaches^23–26^ by providing a system-resolved diagnostic of whether predictions respect both the linearized physical law and the dispersion structure of the experimental record. Because these diagnostics are anchored in published measurements, AEM-based coherence integrates naturally with existing data-validation frameworks for thermophysical properties^27,28^.

**Table 1:** Truth-anchored AEM summary statistics for base and ensemble predictors across all systems. Medians and quantiles are computed from system-level AEM coordinates. Here $d_{0.05}$ and $d_{0.95}$ denote the 5th and 95th percentiles of $d_{\textrm{AEM}}$; $\pi _{<1}=\Pr (d_{\textrm{AEM}}<1)$; and $\pi _{\Delta H<0}=\Pr (\Delta H<0)$.

Model family	$n_{\textrm{pairs}}$	Med. $S_{\textrm{struct}}$	Med. $\Delta H$	Med. $d_{\textrm{AEM}}$	$d_{0.05}$	$d_{0.95}$	$\pi _{<1}$	$\pi _{\Delta H<0}$
GNN (base)	30520	1.18	1.85	2.27	0.34	5.24	0.17	0.05
MCM (base)	30520	1.20	1.87	2.32	0.34	5.22	0.16	0.05
GNN (ensemble)	763	1.25	1.32	1.87	0.27	5.29	0.26	0.09
MCM (ensemble)	763	1.18	1.54	2.00	0.30	5.13	0.23	0.08

### Diagnosing predictor variability and ensembling effects using the AEM

The AEM provides a physics-informed diagnostic for examining how predictive models express structural and stochastic coherence with respect to an experimental reference. Here AEM is used to illustrate how coherence can be resolved, visualized, and interpreted at the level of individual predictors and model families. The specific model families considered serve as representative examples for demonstrating the functionality of AEM, rather than as objects of normative comparative assessment.

**Fig. 4:**
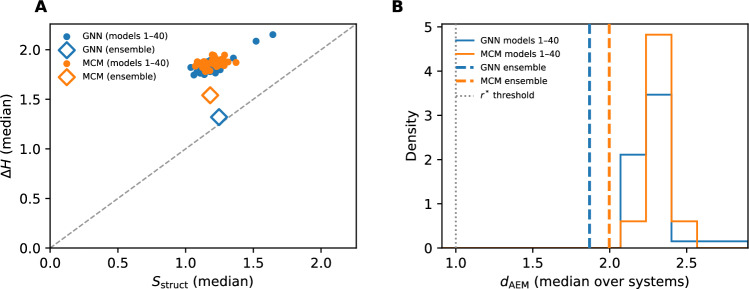
Illustration of predictor variability and ensembling effects using the AEM. (**A**) Median structural deviation $S_{\textrm{struct}}$ versus median entropy lift $\Delta H$ for all base predictors in each illustrative model family (circles) and the corresponding ensemble predictors (diamonds). Ensembles shift predominantly along the stochastic dimension while remaining within the structural support of the base predictors, highlighting variance contraction without structural correction. (**B**) Normalized density of model-level median AEM diagnostics across the forty base predictors in each family. Dashed vertical lines denote the ensemble predictors and indicate how ensembling shifts the typical distance relative to the coherence threshold $d_{\textrm{AEM}}=r^{*}$, without implying convergence toward the ideal origin $(S_{\textrm{struct}},\Delta H)=(0,0)$.

Figure 4 summarizes the distribution of AEM coordinates across forty base predictors within each model family and contrasts them with the corresponding ensemble predictors. Across both families, the base predictors occupy a relatively compact region of the AEM plane (Fig. 4A), indicating broadly similar degrees of structural deviation and stochastic dispersion with respect to the reference mapping. Nevertheless, realization-to-realization differences are resolved by the AEM coordinates and manifest as a nontrivial spread in the joint diagnostic $d_{\textrm{AEM}}$, with median values spanning approximately ${\sim }2.13$–${\sim }2.81$ for one family and ${\sim }2.21$–${\sim }2.43$ for the other (Table 1). This spread reflects intrinsic variability within a fixed modeling paradigm under fixed benchmark conditions.

The ensemble predictors appear as isolated points displaced primarily along the stochastic axis. In both families, ensembling produces a systematic reduction in the entropy lift $\Delta H$ and a corresponding decrease in $d_{\textrm{AEM}}$. This shift is accompanied by an increase in the fraction of systems classified as coherent under the operational criterion $d_{\textrm{AEM}}<r^{*}$ (with $r^{*}=1$ in this work). In the present context, these changes are interpreted as an illustration of how variance contraction alone is represented in the AEM plane, rather than as evidence of structural correction or universally superior modeling strategies.

Importantly, neither the base predictors nor their ensemble counterparts approach the mathematical origin $(S_{\textrm{struct}},\Delta H)=(0,0)$. This origin corresponds to exact agreement in both the linearized mapping and residual dispersion and serves as an idealized limit rather than a physically attainable reference for experimental thermodynamic data or learned predictors. Instead, all predictors remain well separated from this point, while ensemble predictors shift systematically toward smaller joint diagnostics relative to the coherence threshold $d_{\textrm{AEM}}=r^{*}$. Their structural deviations remain within the support spanned by the base predictors, indicating that ensembling reduces stochastic dispersion without eliminating the geometric offset associated with the implied physical mapping.

Figure 4B provides a complementary projection by mapping each predictor to its model-level median $d_{\textrm{AEM}}$. The normalized densities correspond to distributions of base-model medians and visualize internal variability within each family on equal footing. Ensemble predictors appear as reference markers indicating how variance contraction shifts the typical distance relative to $r^{*}$, without implying convergence toward the ideal limit or imposing a total ordering that is invariant to the structural–stochastic decomposition.

Taken together, this analysis demonstrates how AEM functions as a physics-grounded validation lens rather than a single-score ranking metric. By diagnosing structural incompatibility alongside stochastic dispersion and by resolving intra-family variability, AEM provides a geometric language for interpreting predictor behavior in the presence of experimental uncertainty. The examples shown here therefore serve primarily to illustrate the diagnostic capabilities of AEM, which can be applied generically to physics-informed model evaluation in thermodynamics and related domains^23–25^.

### Pairwise coherence across independent experimental sources

To assess the internal consistency of independently reported experimental data governed by the same linearizable thermodynamic law, AEM coordinates were computed for all admissible pairs of experimental sources on their shared domains. Under ideal agreement, such pairs would cluster near the origin $(S_{\textrm{struct}},\Delta H)=(0,0)$ in the AEM plane, reflecting identical least-squares mappings and comparable levels of residual dispersion. In practice, long-standing concerns regarding the reproducibility of thermodynamic measurements, heterogeneity across reported property datasets, and the propagation of cross-source discrepancies into downstream modeling and process design are well documented^8,27,28^, motivating a systematic, physics-informed comparison.

**Fig. 5:**
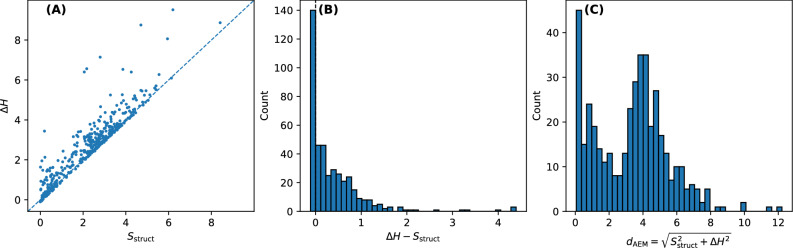
Agreement–Entropy analysis of 424 experimental source pairs. (**A**) Joint distribution of structural deviation $S_{\textrm{struct}}$ and stochastic deviation $\Delta H$. Coherent pairs would cluster near (0, 0); instead, most points occupy a structurally dominated region in which $\Delta H$ increases in concert with $S_{\textrm{struct}}$. (**B**) Distribution of $\Delta H - S_{\textrm{struct}}$, showing a pronounced positive tail corresponding to stochastic disagreement not explained by structural mismatch alone. (**C**) Distribution of composite distances $d_{\textrm{AEM}}=\sqrt{S_{\textrm{struct}}^{2}+\Delta H^{2}}$, with the majority of pairs far from the coherent regime.

Figure 5 shows that the observed experimental landscape departs markedly from this idealized coherent regime. Across all admissible source pairs, the median structural deviation is $S_{\textrm{struct}}=2.32$, while the median stochastic deviation is $\Delta H=2.72$, indicating substantial disagreement both in the inferred linearized mappings and in residual dispersion. A strong positive correlation between the two coordinates ($r=0.93$) is observed; however, this correlation should not be interpreted as evidence of agreement. Instead, it reflects that a large fraction of the entropy lift captured by $\Delta H$ arises from the same mechanism that inflates the restricted–unrestricted residual sum of squares. Such behavior is consistent with scenarios in which systematic biases or inconsistencies in the underlying property data simultaneously distort fitted thermodynamic parameters and amplify residual dispersion upon pooling, as predicted by the geometry of Chow-type comparisons^8,12,13^.

In this regime, the AEM therefore acts predominantly as a structural diagnostic. The experimental pairs align closely with the diagonal geometry associated with slope and intercept mismatch, indicating that cross-source inconsistency is driven primarily by differences in the inferred functional mapping rather than by independent noise effects alone. This observation is consistent with recent analyses showing that apparently minor inconsistencies in thermophysical property data can induce systematic, model-relevant distortions in fitted thermodynamic relationships when propagated through regression-based workflows^8^. At the same time, the finite spread around the diagonal and the pronounced positive tail in $\Delta H - S_{\textrm{struct}}$ demonstrate that stochastic contributions are not uniformly explained by structural mismatch alone. These deviations capture variance inflation and distributional effects that are invisible to single-axis structural statistics and conventional accuracy-based validation metrics.

Panels B and C of Fig. 5 quantify these departures. The distribution of $\Delta H - S_{\textrm{struct}}$ is centered near zero but exhibits a substantial positive tail, confirming that many source pairs accumulate stochastic disagreement beyond that predicted by structural geometry alone. The composite distance $d_{\textrm{AEM}}$ has a median value of 3.62, with approximately $68\,\%$ of pairs satisfying $d_{\textrm{AEM}}>2$ and upper quantiles extending beyond 6. Such magnitudes are incompatible with the assumption of mutually coherent subsets and indicate that, for many systems, independent sources trace systematically different linearized thermodynamic relationships. From a practical standpoint, this finding supports concerns that uncritical aggregation of heterogeneous experimental data can mask physically meaningful inconsistencies while yielding deceptively stable pooled metrics^8^.

Overall, the AEM provides a physically interpretable mapping of inter-source coherence. For the present dataset, disagreement is dominated by structural mismatch, yet the entropy axis reveals additional stochastic dispersion beyond that implied by structural geometry alone. This layered structure of disagreement underscores that experimental inconsistency arises from multiple, overlapping mechanisms rather than from a single dominant source, and highlights the need for physics-informed, variance-aware validation procedures in thermodynamics-guided machine learning and chemical engineering data analysis^23–26^.

## Conclusion

This work introduced a physics-informed validation framework for assessing structural and stochastic coherence between predictive models and experimental data governed by known physical relationships. The AEM combines the restricted–unrestricted regression geometry underlying Chow-type comparisons^12,13^ with an entropy-motivated characterization of residual dispersion rooted in classical information theory^14,15^. By operating on matched comparison domains and diagnosing structural misalignment alongside variance-driven dispersion, AEM provides insight that cannot be obtained from pooled scalar accuracy metrics, which are intrinsically insensitive to violations of governing physical laws^7,9–11^.

Using thermodynamic systems as a canonical physics-governed testbed, we quantified inter-source disagreement in a curated collection of experimental measurements drawn from ILThermo^19,20^. Despite common linearizing transformations, the experimental landscape exhibits substantial structural divergence and stochastic inconsistency across independent reports. These findings complement established thermophysical data-evaluation and quality-assurance efforts^27,28^ by providing a quantitative, model-agnostic characterization of cross-source coherence that is directly interpretable in terms of physical structure and residual disorder.

The framework was further applied to two families of physics-guided predictive models–a graph neural network and a matrix-completion architecture–trained on temperature-dependent activity coefficients^21,22^. Truth-anchored analysis revealed that ensemble predictors systematically reduce stochastic deviation while leaving structural offsets largely unchanged, whereas individual realizations exhibit pronounced variability along both AEM coordinates. This diagnostic distinction clarifies when apparent improvements in conventional validation metrics arise from variance contraction rather than enhanced mechanistic agreement, and it enables system-resolved assessment of model fidelity that is inaccessible to single-score evaluations. In this role, AEM complements recent advances in thermodynamics-consistent and physics-informed learning by supplying a validation lens aligned with physical interpretability^23–26^.

Taken together, the AEM establishes a rigorous and interpretable foundation for physics-informed validation. By quantifying how and why two sources disagree–rather than merely how much their predictions differ–AEM provides a general diagnostic tool applicable to physics-informed machine learning, inter-laboratory consistency studies, and the validation of predictive models and datasets in complex physics-governed systems through physically motivated linearization.

## Supplementary Information

Supplementary Information.

## Data Availability

All raw data generated and analysed during this study, together with all code used to produce the reported results, are openly available in a public GitHub repository at https://github.com/HedayatHaddadi/AEM.
