# Supplementary material for: Physics-informed structural diagnostics of model–data agreement beyond scalar metrics
Source: Sci Rep. 2026 Apr 22;16:18622. doi: 10.1038/s41598-026-49445-8 (PMC13269754; doi:10.1038/s41598-026-49445-8)
Supplement: Supplementary file 1 — Supplementary Information. [file 41598_2026_49445_MOESM1_ESM.pdf]

Supporting Information

Physics-informed structural diagnostics of model–data agreement beyond scalar metrics

Hedayat Haddadi[1], Adam Kloskowski[1], and Piotr Mironowicz[2]

[1]Department of Physical Chemistry, Faculty of Chemistry, Gdańsk University of Technology, 80-233 Gdańsk, Poland
[2]Department of Algorithms and Systems Modelling, Faculty of Electronics, Telecommunications and Informatics, Gdańsk University of Technology, 80-233 Gdańsk, Poland

Table of Contents

- S1. Overview and notation
  - S1.1 Basic objects and notation
  - S1.2 Purpose of the AEM coordinates
- S2. Mathematical framework
  - S2.1 Linearized mapping and overlap geometry
  - S2.2 Structural deviation coordinate
  - S2.3 Entropy-based stochastic deviation
  - S2.4 ANOVA–entropy identity and reinterpretation of F_{Chow}
  - S2.5 AEM coordinates, distance, and model-level summaries
  - S2.6 Complementarity of structural and stochastic diagnostics
- S3. Worked numerical example (overlap gating; anchored and symmetric variants)
- References

S1. Overview and notation

The Agreement–Entropy Map (AEM) provides a general, physics-informed framework for evaluating the coherence between two data sources or predictive mechanisms that are expected to obey a common governing relationship. The framework applies whenever prior physical knowledge permits a linear or linearizable representation between a transformed response variable and an explanatory variable, as commonly encountered in physics-informed machine learning, data-driven scientific discovery, and physics-guided modelling.

Rather than assessing agreement solely through pooled prediction error, AEM evaluates whether two sources encode the same functional mapping implied by the underlying physical law and whether their residual variability is statistically compatible. In this sense, AEM is model-agnostic and domain-independent: it can be applied uniformly to experiment–experiment, model–experiment, and model–model comparisons across physics and engineering disciplines, provided that the comparison is conducted on a shared domain defined by the governing relation.

Operationally, AEM is constructed from least-squares residuals obtained after enforcing the relevant linear or linearized physical constraint. These residuals are used to define two complementary diagnostic coordinates that quantify structural deviation in the inferred mapping and stochastic deviation in residual disorder. Section S2 develops these coordinates rigorously; the purpose of the present section is solely to introduce the notation and conventions used throughout the Supporting Information.

S1.1 Data subsets and overlap domain

Let

$$S_1 = \{(x_i, y_i)\}_{i=1}^{n_1}, \qquad S_2 = \{(x'_j, y'_j)\}_{j=1}^{n_2},$$

denote two subsets of observations in transformed coordinates. Comparisons are made only on their common domain

$$\Omega_{12} = [\max(x_{\min,1}, x_{\min,2}),\ \min(x_{\max,1}, x_{\max,2})],$$

with n denoting the number of retained points.

S1.2 Residuals and variance

For any subset S with n_S retained points, let $\hat{y}$ denote the least-squares fitted values and let

$$r = y - \hat{y}$$

be the corresponding residual vector. Throughout this document all logarithms are natural logarithms, and all residual quantities are computed on the comparison domain explicitly indicated (e.g. Ω_{12} in Section S2.1 or Ω_{glob} in Section S2.5).

The residual sum of squares and the empirical residual variance are written

$$\text{SSR}(S) = \sum_{i=1}^{n_S} r_i^2, \qquad \widehat{\sigma}^2(S) = \frac{1}{n_S} \sum_{i=1}^{n_S} r_i^2.$$

These definitions apply to individual subsets, pooled subsets, and model–reference combinations, with the only requirement that the residuals be evaluated on the same set of abscissae for all quantities that are compared.

S1.3 Restricted and unrestricted fits

On the overlap domain Ω_{12} defined in Section S1.1, two regression constructions are required for the structural and stochastic coordinates developed in Section S2:

- **Unrestricted fit:** separate least-squares regressions are fitted to S_1 and S_2. The combined residual sum of squares is
$$\mathrm{SSR}_u = \mathrm{SSR}(S_1) + \mathrm{SSR}(S_2),$$
where both terms are computed using only the observations lying in Ω_{12}.

- **Restricted fit:** a single pooled regression is fitted to $S_1 \cup S_2$ on Ω_{12}, producing the residual sum of squares
$$\mathrm{SSR}_r = \mathrm{SSR}(S_1 \cup S_2).$$

By construction, SSR_r and SSR_u are always computed from the same $n = n_1 + n_2$ observations. This alignment ensures that ratios such as $\mathrm{SSR}_r/\mathrm{SSR}_u$ and the associated logarithmic transforms used in AEM reflect genuine structural differences rather than differences in sample size or domain.

S2. Mathematical framework

This section develops the Agreement–Entropy Map (AEM) using the notational objects introduced in Section S1. The framework is entirely descriptive: it assigns a pair of diagnostic coordinates to two data-generating mechanisms by applying deterministic functionals to restricted and unrestricted least-squares residuals computed on a matched domain. All logarithms are natural unless stated otherwise, and all residual quantities are evaluated on the specific domain indicated for each comparison.

AEM requires only linear-model geometry, the algebraic structure of restricted–unrestricted regressions, and the variance–entropy functional derived from the Shannon entropy of a Gaussian distribution. No distributional assumptions (Gaussianity, homoscedasticity, independence beyond least-squares consistency) are imposed for the definitions of the coordinates. This stands in contrast to the classical Chow test, whose inferential use depends on Gaussian, common-variance assumptions; the distinction between these conditions is made explicit in Sections S2.3 and S2.4.

Because the stochastic coordinate is an entropy lift defined by a log variance ratio, it may be positive or negative: positive values indicate that pooling inflates residual dispersion, while negative values indicate reduced dispersion relative to the reference. The interpretation of such negative values, including its role in anchor-based and symmetric comparisons, is clarified in Section S2.5. Null calibration for multi-subset comparison, when required, is briefly discussed in Section S2.6.

Section S2.1 introduces the linearized regression setting and the overlap geometry that motivates restricted and unrestricted fits. Section S2.2 defines the structural deviation as the logarithmic ratio of restricted to unrestricted sums of squared residuals. Section S2.3 develops the stochastic deviation using Gaussian plug-in entropies and clarifies the assumptions under which this coordinate

is well defined. Section S2.4 records the algebraic relationship between the structural coordinate and the classical Chow statistic and explains the shift from inferential to geometric interpretation. Section S2.5 formalizes the AEM coordinates for model–reference and symmetric pairwise comparisons and defines the AEM distance. Section S2.6 analyzes the complementarity of the two axes under variance imbalance, mixture structure, and heteroscedasticity, and outlines how symmetric coordinates may be used for consensus assessment among multiple datasets.

S2.1 Linearized mapping and overlap geometry

Many physical systems admit representations that are linear, or approximately linear, after an appropriate transformation motivated by governing equations, symmetries, or dimensional analysis. Such linearizations arise broadly in physics-informed modelling, including but not limited to rate laws, transport relations, equilibrium mappings, response functions, and other constitutive relationships. The Agreement–Entropy Map therefore begins with a general linear or linearizable representation of the form (see Rencher and Schaalje,[1] Ch. 6)

$$y = a + bx + \varepsilon, \tag{1}$$

where x denotes a transformed explanatory variable defined by the governing relation, y is the corresponding transformed response, (a, b) are the parameters encoding the effective physical mapping, and ε is a random error term capturing unresolved variability.

For the algebraic constructions underlying the AEM, only minimal statistical conditions are required:

$$\mathbb{E}[\varepsilon] = 0, \qquad 0 < \mathrm{Var}(\varepsilon) < \infty,$$

with independence across observations. No distributional assumptions are imposed in defining the AEM coordinates. Gaussianity is invoked only when recalling classical inferential results associated with least-squares regression and plays no role in the construction or interpretation of the AEM diagnostics themselves.

Principle of a single functional mapping. When two data subsets describe the same underlying physical or mechanistic system, a linearization such as (1) induces a *unique effective functional mapping* on any shared domain of the explanatory variable x. Let S_1 and S_2 denote two such subsets, with least-squares estimates (a_1, b_1) and (a_2, b_2) obtained on their respective domains. The natural structural null hypothesis is therefore

$$H_0 : (a_1, b_1) = (a_2, b_2),$$

which corresponds to equality of regression coefficients in classical analysis of covariance (ANCOVA) (Rencher and Schaalje,[1] Ch. 16).

Rejection of this null indicates that the two subsets do not admit a common functional representation on their overlap. From a physics-informed perspective, this implies that the inferred governing mapping differs between sources, and that discrepancies cannot be attributed to sampling variability alone. Such structural disagreement signals inconsistency at the level of the physical relationship itself, independent of residual noise or data volume.

Overlap gating and restricted–unrestricted models. Let S_1 and S_2 contain transformed abscissae $x_1, \ldots, x_{n_1}$ and $x'_1, \ldots, x'_{n_2}$, respectively. A meaningful comparison requires restricting attention to the common domain

$$\Omega_{12} = [\max(x_{\min,1}, x_{\min,2}),\ \min(x_{\max,1}, x_{\max,2})],$$

where $x_{\min,i}$ and $x_{\max,i}$ denote the extreme values in each subset. Let n_1 and n_2 denote the numbers of points from each subset that lie in Ω_{12}, with $n = n_1 + n_2$.

On this overlap, two least-squares models encode structural coherence:

- *Unrestricted model:* Each subset is fitted separately, yielding $\mathrm{SSR}_u = \mathrm{SSR}_1 + \mathrm{SSR}_2$ with four free parameters.
- *Restricted model:* A single pooled line (a_r, b_r) is fitted to $S_1 \cup S_2$, yielding SSR_r with two free parameters.

The restriction thus imposes $k = 2$ linear constraints, which is precisely the difference in dimensionality between the unrestricted and restricted models. This constitutes a special case of the general linear hypothesis $H_0 : C\beta = 0$ in linear-model theory (Rencher and Schaalje,[1] Ch. 8), where the matrix C encodes the equality of regression coefficients across groups.

Limitations of pooled accuracy and classical reproducibility metrics. Pointwise accuracy measures such as RMSE, MAE, and R^2 depend solely on the pooled residual vector $r = y - \hat{y}$ from the restricted fit, that is, on SSR_r or on $|r_i|$. Because these metrics do not incorporate the unrestricted quantity SSR_u, they cannot determine whether two subsets obey the same functional mapping $(a_1, b_1) = (a_2, b_2)$.

This limitation follows directly from least-squares geometry. If $\hat{b}_i$ is the slope estimated from subset S_i and b_r is the pooled slope, then (Rencher and Schaalje,[1] Ch. 7)

$$\mathrm{SSR}_i(b_r) = \mathrm{SSR}_i(\hat{b}_i) + (\hat{b}_i - b_r)^2\, S_{xx,i}, \qquad S_{xx,i} = \sum_{x \in S_i} (x - \bar{x}_i)^2, \tag{2}$$

showing that the penalty for enforcing an incorrect slope increases quadratically with the deviation $\hat{b}_i - b_r$. When the x-spread within a subset is small, even substantial differences in slope yield only small increases in the pooled SSR_r, causing RMSE, MAE, and R^2 to remain deceptively favorable.

A parallel issue occurs in classical reproducibility metrics used in interlaboratory comparisons. Variance-component models (repeatability s_r, reproducibility s_R), intraclass correlation coefficients (ICC), Bland–Altman limits of agreement, and concordance correlation coefficients (CCC) each operate on the empirical distribution of responses $\{y_{ij}\}$ but contain no mechanism for detecting differences in the underlying functional law $y = a + bx$. For example, the standard random-effects formulation

$$y_{ij} = \mu + L_i + \epsilon_{ij}, \qquad L_i \sim \mathcal{N}(0, \sigma_L^2),\ \epsilon_{ij} \sim \mathcal{N}(0, \sigma_r^2),$$

separates between- and within-laboratory variability, yet it cannot detect differences in slope. ICC exhibits similar insensitivity: it remains high even when laboratories report different (a, b) values, provided the noise is small or the range of x is narrow. Bland–Altman differences satisfy

$$d_j = y_{1j} - y_{2j} = (a_1 - a_2) + (b_1 - b_2)\, x_j,$$

which is x-dependent whenever slopes differ, yet the method does not provide a test for equality of (a_1, b_1) and (a_2, b_2). CCC behaves analogously: mean and scale discrepancies are penalized, but changes in regression slope are largely invisible.

These approaches focus on numerical agreement between measurements rather than on agreement of the underlying functional mapping. As a result they cannot determine whether two subsets share the same linear or linearizable law. Detecting structural coherence requires a restricted–unrestricted comparison such as the Chow statistic, whereas detecting stochastic coherence requires a distributional diagnostic such as the entropy axis of AEM. Together, these two components form the basis for the structural–stochastic geometry developed in subsequent sections.

S2.2 Structural deviation coordinate

Classical Chow statistic. When two data subsets are each well represented by a linear or linearizable relationship, a basic question is whether they share the same regression parameters. Under the classical linear–model assumptions—independent, zero-mean Gaussian errors with a common variance under the null hypothesis of a shared mapping—the comparison is performed using the Chow statistic[1;2]

$$F_{\text{Chow}} = \frac{(\text{SSR}_r - \text{SSR}_u)/k}{\text{SSR}_u/(n-2k)}, \tag{3}$$

where SSR_u and SSR_r denote the unrestricted and restricted sums of squared residuals, $k = 2$ is the number of restrictions imposed by the pooled model, and $n = n_1 + n_2$ is the total number of retained points on the overlap domain. Inference based on (3) requires both subsets to contain enough observations to estimate separate regressions and for the unrestricted fit to retain positive residual degrees of freedom; for simple straight-line models, $n_i \geq 3$ ensures $n - 2k > 0$. Under these Gaussian–homoscedastic conditions, $F_{\text{Chow}} \sim F(k, n-2k)$, and the associated p-values are valid.

In the AEM framework, these inferential assumptions are not imposed: only the algebraic structure of the restricted–unrestricted SSR comparison is retained, and (3) is used descriptively rather than as a hypothesis test.

In the AEM framework, however, the Chow statistic is not used for inference. Only its algebraic connection to the restricted–unrestricted sum-of-squares ratio is retained, enabling a descriptive and domain-agnostic structural metric.

Structural log–SSR ratio as a descriptive coordinate. The core structural quantity is the ratio $\text{SSR}_r/\text{SSR}_u$, which compares the loss of fit incurred by enforcing a single functional law to the fit achieved when subsets are allowed separate parameters. Because the restricted model is nested within the unrestricted model, the classical geometry of least squares guarantees (Rencher and Schaalje,[1] Ch. 8)

$$\text{SSR}_r \geq \text{SSR}_u,$$

with equality if and only if the constraints $(a_1, b_1) = (a_2, b_2)$ are fully compatible with the data on the overlap domain.

AEM expresses this structural deviation on a logarithmic scale:

$$S_{\text{struct}} = \frac{1}{2} \ln\left(\frac{\text{SSR}_r}{\text{SSR}_u}\right), \tag{4}$$

a quantity that is well defined whenever $0 < \mathrm{SSR}_u < \infty$ and strictly increasing in the SSR ratio. The factor of $1/2$ ensures direct compatibility with the entropy-based formulation introduced in Section S2.3, allowing both axes of AEM to be expressed in the same natural (logarithmic) unit. The coordinate S_{struct} is nonnegative, dimensionless, and equals zero precisely when pooled and separate regressions yield identical residual dispersion.

The degrees-of-freedom normalization in (3) is needed only for inferential calibration under an F distribution. When hypothesis testing is not the goal, the mapping from F_{Chow} to the SSR ratio is strictly monotone and adds no information beyond $\mathrm{SSR}_r/\mathrm{SSR}_u$ itself. Thus S_{struct} in (4) is the natural structural coordinate for descriptive diagnostics.

Sample-size invariance. Because both SSR_r and SSR_u are computed from the same n observations in the overlap domain Ω_{12}, trivial changes in sample size scale both quantities proportionally and cancel in the ratio. Consequently, deviations of $\mathrm{SSR}_r/\mathrm{SSR}_u$ from unity reflect genuine structural misalignment between the pooled and separate fits rather than differences in the number of measurements. This makes the structural coordinate robust across datasets with heterogeneous sampling density and supports its use in multi-system and multi-source comparisons.

S2.3 Entropy-based stochastic deviation

To characterize stochastic disagreement between two data-generating mechanisms, AEM employs an entropy-based coordinate that measures how residual dispersion changes when different sources or models are compared on a shared domain. The construction relies solely on the variance of residuals and the information-theoretic observation that, among all distributions with a fixed variance, the Gaussian has the largest differential entropy. This provides a natural, domain-agnostic variance-to-entropy functional without assuming that the underlying residuals are themselves Gaussian.[3]

Gaussian plug-in entropy. For a zero-mean Gaussian distribution $\mathcal{N}(0, \sigma^2)$ the classical Shannon entropy is

$$H(\mathcal{N}(0, \sigma^2)) = \frac{1}{2} \ln(2\pi e \sigma^2), \tag{5}$$

a well-known result due to Shannon[4] and Cover and Thomas.[3] Because this entropy is maximal among all distributions with variance σ^2 (Cover and Thomas, Ch. 8), any residual distribution r with the same variance satisfies

$$H(r) \;\leq\; H(\mathcal{N}(0, \sigma^2)),$$

with equality only in the Gaussian case.

In AEM, (5) is used as a variance-to-entropy mapping. Given an empirical variance $\widehat{\sigma}^2$ of a residual ensemble, the plug-in entropy

$$H_G(\widehat{\sigma}^2) := \frac{1}{2} \ln(2\pi e \widehat{\sigma}^2)$$

is assigned. This quantity depends only on the second moment of the residuals, provides an upper bound on the true entropy, and requires no distributional modeling of the residual shape.

Entropy lift between two residual ensembles. Let A and B denote two sets of residuals evaluated at the same n points, with sums of squares SSR_A and SSR_B. Using the maximum-likelihood variance estimators $\widehat{\sigma}_A^2 = \mathrm{SSR}_A/n$ and $\widehat{\sigma}_B^2 = \mathrm{SSR}_B/n$, their plug-in entropies are

$$H_A = \frac{1}{2}\ln(2\pi e) + \frac{1}{2}\ln\!\left(\frac{\mathrm{SSR}_A}{n}\right), \qquad H_B = \frac{1}{2}\ln(2\pi e) + \frac{1}{2}\ln\!\left(\frac{\mathrm{SSR}_B}{n}\right).$$

The entropy lift,

$$\Delta H(A,B) := H_A - H_B = \frac{1}{2}\ln\!\left(\frac{\mathrm{SSR}_A}{\mathrm{SSR}_B}\right), \tag{6}$$

is purely algebraic and depends only on the ratio of sums of squares; the common constant $\frac{1}{2}\ln(2\pi e/n)$ cancels exactly.

When A and B correspond to the restricted and unrestricted regressions, (6) shows that the structural log–SSR ratio,

$$\Delta H(r,u) = \tfrac{1}{2}\ln(\mathrm{SSR}_r/\mathrm{SSR}_u) = S_{\mathrm{struct}},$$

is itself an entropy lift. Thus, both axes of AEM ultimately arise from the same log-ratio structure, applied in different residual geometries.

Assumptions for the entropy axis. For the stochastic deviation $\Delta H(A,B)$ to be meaningful in general physics-governed settings admitting linear or locally linearizable mappings, only the following mild conditions are required:

(E1) **Finite, nondegenerate variance.** Both residual ensembles have variances in $(0,\infty)$.

(E2) **Common physical scale.** Residuals for A and B are expressed in the same transformed coordinates, corresponding to the same physical observable or response variable.

(E3) **Centering by fitted mappings.** Residuals are computed relative to the respective least-squares representations of the governing relationship, so that systematic offsets in the functional mapping do not contaminate variance comparisons.

(E4) **Entropy as a variance functional only.** The Gaussian entropy expression is employed solely as a variance-to-entropy mapping; no distributional assumptions on the residuals are required.

Under (E1)–(E4), $\Delta H(A,B)$ depends only on the ratio of residual dispersions and is therefore well defined for all finite, positive values of SSR_A and SSR_B.

Identifiability of the entropy lift. If A and B have $0 < \mathrm{SSR}_A, \mathrm{SSR}_B < \infty$ on the same n points, then:

1. $\Delta H(A,B)$ is finite and well defined.
2. For fixed SSR_B, the mapping $\mathrm{SSR}_A \mapsto \Delta H(A,B)$ is strictly increasing and injective.
3. For any $\Delta H \in \mathbb{R}$ and fixed SSR_B, there exists a unique $\mathrm{SSR}_A = \mathrm{SSR}_B e^{2\Delta H}$.

Thus, the entropy lift is fully determined by the SSR ratio and uniquely encodes it.

If h_A and h_B are the true differential entropies of the two residual distributions, the maximum-entropy property of the Gaussian gives

$$h_A \le H_A, \qquad h_B \le H_B,$$

with equality only when both distributions are Gaussian. Therefore the true entropy difference satisfies

$$\Delta H_{\text{true}} = h_A - h_B \le \Delta H(A, B), \tag{7}$$

so the plug-in entropy lift is always a conservative (upper-bound) measure of stochastic disorder.

Corollary S2.1 (Characterization of $\Delta H = 0$). For residual ensembles A and B with positive, finite sums of squares,

$$\Delta H(A, B) = 0 \iff \mathrm{SSR}_A = \mathrm{SSR}_B.$$

Thus the entropy lift vanishes exactly when pooling or constraining the fits leaves the empirical second moment unchanged.

Remark S2.1 (Variance-only sensitivity). Because $H_G(\widehat{\sigma}^2)$ depends solely on the variance, $\Delta H(A, B)$ captures only second-moment differences. Consequently, two residual distributions may differ substantially in shape (e.g. skewness, heavy tails, or multimodality) yet share the same variance; in such cases the entropy lift defined in (6) is identically zero by construction. In practice, many cross-source and cross-regime comparisons exhibit variance mismatch and/or domain-dependent heteroscedasticity, in which case ΔH provides an immediate, dimensionless diagnostic of variance-driven stochastic incoherence. Shape-driven discrepancies are not targeted by ΔH and must be assessed using complementary diagnostics if required.

Remark S2.2 (Nonparametric entropy estimators). Alternative estimators such as nearest-neighbor or kernel-based entropy estimators can approximate the true entropy of highly skewed or heavy-tailed distributions, but require larger sample sizes and smoothing parameters. The Gaussian plug-in is attractive because it is defined for any distribution with finite variance and depends only on a readily measurable second moment. In AEM it serves as a robust variance-driven diagnostic rather than an estimator of the true entropy $h(\cdot)$.

S2.4 ANOVA–entropy identity and reinterpretation of F_{Chow}

The Chow statistic provides a classical mechanism for assessing whether two linear subsets share a common regression law. Its inferential interpretation requires strong assumptions, but its algebraic structure is entirely general. Beginning from the definition in (3),

$$F_{\text{Chow}} = \frac{(\mathrm{SSR}_r - \mathrm{SSR}_u)/k}{\mathrm{SSR}_u/(n-2k)} = \frac{n-2k}{k} \, \frac{\mathrm{SSR}_r - \mathrm{SSR}_u}{\mathrm{SSR}_u},$$

one may solve explicitly for the ratio of restricted to unrestricted sums of squares:

$$\frac{\mathrm{SSR}_r}{\mathrm{SSR}_u} = 1 + \frac{k}{n-2k} F_{\text{Chow}}. \tag{8}$$

This relation is an identity: it holds for any realized residual vectors for which least-squares fitting is well defined and for any $n-2k > 0$. No distributional assumptions—Gaussianity, homoscedasticity,

or independence— are required for (8). Hence the connection between F_{Chow} and the structural SSR ratio extends broadly to linear or linearizable models across scientific and engineering disciplines.

Substituting (8) into the entropy-lift expression (6) with $A = r$ and $B = u$ yields

$$S_{\text{struct}} = \frac{1}{2}\ln\!\left(1 + \frac{k}{n-2k}F_{\text{Chow}}\right), \tag{9}$$

with inverse mapping

$$F_{\text{Chow}} = \frac{n-2k}{k}\left(e^{2S_{\text{struct}}} - 1\right).$$

Thus S_{struct} and F_{Chow} are strictly monotone transformations of the same underlying structural quantity—the restricted–unrestricted SSR ratio. AEM adopts the logarithmic form because it expresses this structural deviation on the same entropy scale used for stochastic diagnostics, enabling direct geometric comparison between the two axes.

Interpretation under the classical Chow-test assumptions. When the classical inference assumptions hold (independent errors, common variance across subsets, Gaussian noise under H_0),[1;2] the distributional result $F_{\text{Chow}} \sim F(k, n-2k)$ justifies hypothesis testing for equality of regression coefficients. In this idealized regime, (9) shows that the AEM structural coordinate is merely a reparameterized version of the F statistic and does not add inferential power beyond the classical test.

Outside this narrow setting—which includes nearly all real experimental applications—errors may be heteroscedastic, non-Gaussian, cross-correlated, or source-dependent. In such cases F_{Chow} no longer follows an F distribution, its p-values are not meaningful, and inference based on the classical test is invalid. However, the algebraic identity (8) remains fully valid, and the restricted–unrestricted SSR ratio continues to quantify structural misalignment. AEM therefore treats F_{Chow} as a descriptive index and uses S_{struct} as the principal coordinate because it depends only on realized SSR values and aligns naturally with the entropy-based stochastic axis.

Remark S2.3 (Gaussian limit and collapse of SSR and variance). Under i.i.d. Gaussian errors with a common variance, the empirical variance and the residual sum of squares encode equivalent information: $\widehat{\sigma}^2 = \text{SSR}/n$, and the Gaussian plug-in entropy $H = \frac{1}{2}\ln(2\pi e\,\widehat{\sigma}^2)$ is a monotone reparameterization of SSR. In this special limit, structural and stochastic diagnostics collapse onto a single degree of freedom, and the two AEM axes become algebraically coupled.

Outside this regime—when residuals exhibit heteroscedasticity, mixture structure, heavy tails, skewness, or instrument-specific noise—SSR no longer captures all aspects of the residual distribution. The stochastic variance ratio and the structural SSR ratio therefore diverge, revealing independent modes of incoherence. Consequently, the two-axis structure of AEM is essential for real experimental and modeling scenarios where Gaussianity and homoscedasticity rarely hold.

S2.5 AEM coordinates, distance, and model-level summaries

AEM coordinates for model–reference coherence. When a predictive mechanism M is compared with a trusted reference subset R on a shared evaluation domain Ω_{glob}, disagreement may arise through two geometrically distinct channels: (i) the functional form required to fit the data on the overlap domain (structural deviation), and (ii) the distributional properties of residuals when M and R are pooled on the global domain (stochastic deviation). AEM encodes these two mechanisms

as logarithmic entropy lifts, thereby placing structural and stochastic deviations on the same natural (ln–based) scale and permitting joint geometric interpretation.

The construction requires only that the underlying relationship be linear or linearizable on the overlap and that residuals share common physical units and finite variance. No Gaussian or common-variance assumptions are imposed. Under these mild conditions, the stochastic coordinate may be either positive or negative:

$\Delta H > 0$ indicates dispersion inflation, $\Delta H < 0$ indicates dispersion reduction relative to the anchor.

Negative stochastic values arise naturally in truth-anchored scenarios where the reference is exceptionally stable and the model introduces less residual variability on the pooled domain. The anchored and symmetric variants discussed below formalize these situations.

Although thermodynamic systems serve as the primary case study in this work, where well-established linearizable governing laws enable transparent interpretation, the formalism applies broadly to scientific and engineering contexts in which a shared physical relationship can be represented by a linear or locally linearizable mapping on a common domain.

- **Structural coordinate.** On the overlap of M and R, we fit a restricted pooled regression and an unrestricted pair of regressions. The resulting structural coordinate is

$$x_M := S_{\text{struct}}(M) = \frac{1}{2}\ln\left(\frac{\text{SSR}_r(M)}{\text{SSR}_u(M)}\right).$$

  This measures the entropy lift incurred by enforcing a shared linear mapping. It equals zero when pooled and separate regressions give identical dispersion and increases monotonically as structural misalignment grows.

- **Stochastic coordinate.** Across the full domain Ω_{glob}, the reference R is treated as the anchor. Let $\widehat{\sigma}_R^2$ denote the variance of residuals for R, and $\widehat{\sigma}_S^2$ the variance after pooling residuals from M and R. The stochastic coordinate is then the entropy lift

$$y_M := \Delta H(M) = \frac{1}{2}\ln\left(\frac{\widehat{\sigma}_S^2}{\widehat{\sigma}_R^2}\right),$$

  which captures inflation (or reduction) of residual dispersion induced by incorporating model predictions. It detects heteroscedasticity, mixture structure, or any change in residual disorder relative to the reference.

The pair (x_M, y_M) therefore represents the structural and stochastic entropy lifts produced by substituting the model for the reference. Both quantities are expressed in the same natural unit (nats), and the origin $(0, 0)$ corresponds to perfect structural and stochastic coherence.

Alternative stochastic coordinates: anchored and symmetric. When a single subset R is designated as the most reliable source of residual information (e.g. truth-anchored evaluation), stochastic deviation is naturally quantified by the anchored entropy lift

$$\Delta H_{\text{anch}}(M \mid R) = \frac{1}{2}\ln\left(\frac{\widehat{\sigma}_{R\cup M}^2}{\widehat{\sigma}_R^2}\right), \tag{10}$$

which measures how much the model degrades or improves the dispersion of the reference when the two residual ensembles are pooled. The condition $\Delta H_{\text{anch}} = 0$ indicates that pooling the model with the reference leaves the variance of the anchor unchanged.

When no subset can be identified *a priori* as more reliable, symmetry is essential. The symmetric entropy lift

$$\Delta H_{\text{sym}}(M, R) = \frac{1}{2} \ln\left(\frac{\widehat{\sigma}^2_{R\cup M}}{\sqrt{\widehat{\sigma}^2_R\, \widehat{\sigma}^2_M}} \right), \tag{11}$$

compares the pooled variance to the geometric mean of the individual variances and is invariant under exchanging M and R. This makes it suitable for clustering and consensus analysis across many experimental subsets.

Whenever both individual variances are available, the anchored and symmetric forms satisfy the identity

$$\Delta H_{\text{sym}}(M, R) = \Delta H_{\text{anch}}(M \mid R) - \frac{1}{4} \ln\left(\frac{\widehat{\sigma}^2_M}{\widehat{\sigma}^2_R} \right), \tag{12}$$

showing that the symmetric lift equals the anchored lift corrected by the relative noise scales of M and R. In the present work the anchored form (10) is used for model–reference comparisons, while (11) is employed when no external reference is available.

Remark S2.5 (Pooling does not impose distributional assumptions). The structural coordinate compares restricted and unrestricted least-squares fits on the overlap domain, while the stochastic coordinate uses empirical variances on the full domain. These constructions require only that (i) the relationship is linearizable on the overlap, (ii) residuals share common units, and (iii) second moments are finite. Neither coordinate assumes Gaussianity, independence, equal noise structure, or any specific residual distribution. Pooling serves only as a diagnostic—revealing how dispersion changes when two residual ensembles are combined—and does not impose any probabilistic model on their joint distribution.

AEM distance (raw entropy geometry). Because x_M and y_M are expressed in the same logarithmic entropy unit, they can be combined without scaling. The Euclidean norm

$$d_{\text{AEM}}(M) = \sqrt{x_M^2 + y_M^2}$$

provides a unified measure of joint structural and stochastic deviation. Models with small $d_{\text{AEM}}(M)$ lie near the coherence point, while large values indicate substantial departure along one or both axes.

Model-level performance across multiple systems. Suppose a model m is evaluated across systems $j = 1, \ldots, J$, each admitting a linear or linearizable representation. Let $(x_{j,m}, y_{j,m})$ denote the corresponding AEM coordinates and

$$d_{j,m} := \sqrt{x_{j,m}^2 + y_{j,m}^2}$$

their associated distances. Performance over the full collection of systems can be summarized using functionals of $\{d_{j,m}\}$.

A measure of typical behavior is given by the median

$$D_m^{\mathrm{med}} = \mathrm{median}_j(d_{j,m}),$$

while sensitivity to challenging or low-coherence systems may be assessed using upper quantiles

$$D_m^{\mathrm{tail}}(q) = \mathrm{q}_q(d_{j,m}), \qquad q \in (0.5, 1),$$

for example $q = 0.90$ or 0.95. A complementary notion of global reliability is given by the coverage rate

$$\pi_m(r^\star) = \frac{1}{J}\sum_{j=1}^{J} \mathbf{1}\{d_{j,m} \le r^\star\},$$

which reports the fraction of systems for which the model lies within a specified tolerance radius $r^\star$ in the AEM plane.

For direct pairwise comparison between two predictive models m_1 and m_2, the differences

$$\Delta d_j = d_{j,m_1} - d_{j,m_2}$$

identify systems on which one model is closer to coherence than the other. The proportion of systems with $\Delta d_j < 0$ yields a transparent win–loss score, complementing aggregate measures such as D_m^{med} or $\pi_m(r^\star)$.

S2.6 Complementarity of structural and stochastic diagnostics

The structural and stochastic AEM coordinates capture complementary aspects of regression agreement. The structural axis measures the geometric misfit between restricted and unrestricted regression hyperplanes on the overlap domain, whereas the entropy axis measures the inflation of residual dispersion induced by pooling a reference with another subset or model on the global domain. These coordinates are not mathematically orthogonal: structural deviations typically introduce between-group dispersion and can therefore increase ΔH, while stochastic heterogeneity can inflate the pooled variance without altering the fitted slopes or intercepts. They are, however, genuinely non-redundant. Structural incoherence may arise with only minor changes in pooled variance, and stochastic incoherence can appear even when the mean function is well aligned.

Variance imbalance. If two subsets share the same linear law (the same (a, b) in expectation) but exhibit different residual variances, $\sigma_1^2 \neq \sigma_2^2$, the Chow statistic is insensitive to this heteroscedasticity because it targets only coefficient differences under a common-variance Gaussian model.[2] Pooling such subsets nevertheless increases the entropy of the residual distribution. For equal subset sizes and equal means, the pooled variance is

$$\sigma_{\mathrm{mix}}^2 = \frac{\sigma_1^2 + \sigma_2^2}{2},$$

with Gaussian plug-in entropy

$$H_{\text{mix}} = \frac{1}{2}\ln(2\pi e\sigma_{\text{mix}}^2).$$

The average per-sample entropy of the separate subsets is

$$\overline{H} = \frac{1}{4}\ln(2\pi e\sigma_1^2) + \frac{1}{4}\ln(2\pi e\sigma_2^2).$$

Their difference is

$$H_{\text{mix}} - \overline{H} = \frac{1}{2}\ln\left(\frac{\sigma_1^2 + \sigma_2^2}{2\sigma_1\sigma_2}\right) \geq 0, \qquad (13)$$

with equality only when $\sigma_1^2 = \sigma_2^2$. Thus, even when the structural comparison accepts coherence of coefficients, the entropy axis reveals variance imbalance in the pooled residuals.

Shape effects and mixture structure. Residuals originating from distinct subsets may combine into a pooled distribution that deviates from Gaussianity, for example through skewness, heavy tails, or multimodality. Let r_{pool} denote the pooled residuals and $H(r_{\text{pool}})$ their true differential entropy. Let $H_G(\widehat{\sigma}_{\text{pool}}^2)$ denote the entropy of the zero-mean Gaussian distribution with the same empirical variance $\widehat{\sigma}_{\text{pool}}^2$. Because the Gaussian maximizes entropy among all distributions with fixed variance (Cover and Thomas,[3] Ch. 8),

$$H(r_{\text{pool}}) \;\leq\; H_G(\widehat{\sigma}_{\text{pool}}^2),$$

with equality if and only if r_{pool} is itself Gaussian. It is therefore formally correct to write

$$H_G(\widehat{\sigma}_{\text{pool}}^2) = H(r_{\text{pool}}) + \Delta H_{\text{shape}}, \qquad \Delta H_{\text{shape}} \geq 0,$$

where ΔH_{shape} represents the entropy deficit associated with non-Gaussian shape at fixed variance.

Importantly, the Agreement–Entropy Map does *not* attempt to estimate $H(r_{\text{pool}})$ or ΔH_{shape} directly. The stochastic coordinate in AEM is defined exclusively through the Gaussian plug-in entropy and therefore responds only to changes in the second moment. As a result, shape effects are detected by AEM *only insofar as they induce a change in variance*. Non-Gaussian residual mixtures with exactly matched variance are, by construction, indistinguishable from Gaussian residuals in the AEM stochastic coordinate.

In practical multi-source datasets—such as measurements acquired across different instruments, protocols, or laboratories—mixture structure is frequently accompanied by variance inflation or domain-dependent heteroscedasticity. In such cases, the entropy lift captures the resulting increase in dispersion without attributing it to shape per se. Restricted–unrestricted SSR contrasts alone cannot isolate these effects, while the stochastic AEM axis provides a complementary variance-based diagnostic of stochastic incoherence.

Heteroscedasticity across the domain. Residual variability may also change with the independent variable or with experimental conditions (temperature, pressure, composition, flow rate, etc.). Even if two subsets have comparable marginal variances, differences in $\text{Var}(r(x))$ as a function of x can cause the pooled residuals to exhibit increased dispersion. For instance, one data source may

show nearly constant scatter while another exhibits error "fanning out" at large x. Pooling such subsets produces a residual distribution whose variance exceeds the simple average of the subset-specific variances, thereby increasing the entropy lift. The structural SSR-based comparison, which aggregates squared residuals without resolving their x-dependence, remains largely insensitive to this coupling between variance and domain.

Local structural drift without global variance inflation. Conversely, two subsets may display small but systematic differences in slope or offset confined to a narrow overlap region. This local structural drift can yield a nonzero restricted–unrestricted SSR contrast while leaving the global pooled variance nearly unchanged. In such a scenario, the structural comparison detects a mean-function misalignment, whereas the pooled-variance entropy lift remains small. This illustrates that stochastic diagnostics alone cannot resolve subtle structural deviations that do not materially inflate global variance and motivates retaining a dedicated structural axis in the Agreement–Entropy Map.

Independence and complementarity of variance ratios. The stochastic entropy lift in AEM,

$$\Delta H(M) = \tfrac{1}{2}\ln(\widehat{\sigma}_S^2/\widehat{\sigma}_R^2),$$

depends on the variance of the pooled residuals and the variance of the reference subset. These quantities are derived from a different residual geometry than SSR_r and SSR_u and cannot be reconstructed from the restricted and unrestricted fits alone. Conversely, the structural SSR ratio $\mathrm{SSR}_r/\mathrm{SSR}_u$ does not determine the pooled-to-anchor variance ratio $\widehat{\sigma}_S^2/\widehat{\sigma}_R^2$. Hence:

- Structural coherence on the overlap domain (small $\mathrm{SSR}_r/\mathrm{SSR}_u$) does not preclude stochastic incoherence on the global domain (large $\widehat{\sigma}_S^2/\widehat{\sigma}_R^2$), and
- Stochastic coherence (small $\widehat{\sigma}_S^2/\widehat{\sigma}_R^2$) does not guarantee agreement of separate regressions on the overlap.

The two-dimensional AEM coordinate system is therefore essential for resolving distinct mechanisms by which experimental subsets or model predictions may fail to agree.

Remark S2.6 (Pooling experimental subsets without a designated anchor). In many applications several experimental subsets are available but none can be identified *a priori* as a trusted reference. In such cases the AEM construction can be extended by treating every subset symmetrically.

For any pair of subsets (A, B) with a common domain, a structural coordinate can be defined using the restricted–unrestricted SSR ratio

$$S_{\mathrm{struct}}(A, B) = \tfrac{1}{2}\ln\left(\frac{\mathrm{SSR}_r(A \cup B)}{\mathrm{SSR}_u(A, B)}\right),$$

and a stochastic coordinate can be defined using the symmetric variance lift

$$\Delta H(A, B) = \tfrac{1}{2}\ln\left(\frac{\widehat{\sigma}^2_{A\cup B}}{\sqrt{\widehat{\sigma}_A^2\,\widehat{\sigma}_B^2}}\right),$$

which measures dispersion inflation upon pooling relative to the geometric mean of the individual variances.

After suitable null calibration, these pairwise coordinates yield a two-dimensional distance

$$d(A,B) = \sqrt{\tilde{x}_{A,B}^2 + \tilde{y}_{A,B}^2}$$

for all pairs of subsets. Standard multivariate and graph-based tools—hierarchical clustering, spectral clustering, multidimensional scaling, or consensus-network methods—may then be applied to this distance structure. Such analyses identify coherent clusters of experimental subsets, highlight outliers, and reveal which subsets exhibit the strongest internal consensus. Subsets with minimal average distance to all others provide the most self-consistent representation of the ensemble and can therefore function as data-driven anchors when no external reference exists.

S3. Worked numerical example (overlap gating; anchored and symmetric variants)

This section provides a fully explicit numerical illustration of the Agreement–Entropy Map (AEM) construction, using the definitions and conventions of Sections S1–S2 and matching the implementation used throughout this work. All quantities are computed on the overlap domain, and empirical residual variances are evaluated as unbiased sample variances when degrees of freedom permit.

Setup and overlap gating. Consider two sources measuring the same system after a van't Hoff linearization,

$$\ln K = a + b\,\frac{1}{T} + \varepsilon, \qquad x = \frac{1}{T} > 0.$$

Each source reports seven transformed observations (x, y):

Data.

$$\begin{aligned}
\text{Source 1:}\quad & (0.0020, 1.55),\ (0.0025, 1.95),\ (0.0030, 2.55),\ (0.0035, 3.10),\\
& (0.0040, 3.45),\ (0.0045, 3.90),\ (0.0050, 4.10),\\
\text{Source 2:}\quad & (0.0022, 1.80),\ (0.0025, 1.62),\ (0.0030, 2.72),\ (0.0035, 2.95),\\
& (0.0040, 3.62),\ (0.0045, 3.85),\ (0.0052, 4.20).
\end{aligned}$$

The maximal overlap interval is

$$\Omega = [x_{\min}, x_{\max}], \qquad x_{\min} = 0.0022, \quad x_{\max} = 0.0050.$$

Restricting to Ω retains $n_1 = 6$ points from Source 1 and $n_2 = 6$ points from Source 2, for a pooled total $n = n_1 + n_2 = 12$.

Step 1: unrestricted fits on Ω. Separate least-squares regressions on the retained points give

$$(\hat{a}_1, \hat{b}_1) = (-0.0714,\ 866), \qquad (\hat{a}_2, \hat{b}_2) = (-0.540,\ 1005),$$

with residual sums of squares

$$\mathrm{SSR}_1 = 0.0749, \qquad \mathrm{SSR}_2 = 0.2388, \qquad \mathrm{SSR}_u = 0.3137.$$

Step 2: restricted pooled fit on Ω. Pooling all retained observations and enforcing a single van't Hoff mapping yields

$$(\hat{a}_r, \hat{b}_r) = (-0.297,\ 928),$$

with pooled residual sum of squares

$$\mathrm{SSR}_r = 0.3548.$$

Step 3: structural deviation. The structural AEM coordinate is

$$S_{\mathrm{struct}} = \tfrac{1}{2}\ln\left(\frac{\mathrm{SSR}_r}{\mathrm{SSR}_u}\right) = \tfrac{1}{2}\ln\left(\frac{0.3548}{0.3137}\right) = 0.0616.$$

Step 4: residual variances (unbiased sample variance). Consistent with the implementation and Section S1.2, residual variances are computed as unbiased sample variances:

$$\widehat{\sigma}_1^2 = \frac{\mathrm{SSR}_1}{n_1 - 1} = \frac{0.0749}{5} = 0.01497, \qquad \widehat{\sigma}_2^2 = \frac{\mathrm{SSR}_2}{n_2 - 1} = \frac{0.2388}{5} = 0.04777,$$

$$\widehat{\sigma}_{\cup}^2 = \frac{\mathrm{SSR}_r}{n - 1} = \frac{0.3548}{11} = 0.03226.$$

Step 5: stochastic deviation. *(i) Anchored entropy lift.* Designating Source 1 as the reference R,

$$\Delta H_{\mathrm{anch}} = \tfrac{1}{2}\ln\left(\frac{\widehat{\sigma}_{\cup}^2}{\widehat{\sigma}_1^2}\right) = \tfrac{1}{2}\ln\left(\frac{0.03226}{0.01497}\right) = 0.384.$$

(ii) Symmetric entropy lift. Treating both sources symmetrically,

$$\Delta H_{\mathrm{sym}} = \tfrac{1}{2}\ln\left(\frac{\widehat{\sigma}_{\cup}^2}{\sqrt{\widehat{\sigma}_1^2\widehat{\sigma}_2^2}}\right) = \tfrac{1}{2}\ln\left(\frac{0.03226}{\sqrt{0.01497 \times 0.04777}}\right) = 0.0938.$$

Step 6: joint AEM distances. The resulting joint diagnostics are

$$d_{\mathrm{AEM,anch}} = \sqrt{S_{\mathrm{struct}}^2 + \Delta H_{\mathrm{anch}}^2} = 0.389, \qquad d_{\mathrm{AEM,sym}} = \sqrt{S_{\mathrm{struct}}^2 + \Delta H_{\mathrm{sym}}^2} = 0.112.$$

Remark S3.1 (consistency with implementation). This worked example uses unbiased sample variances ($\mathrm{SSR}/(n-1)$), consistent with the numerical implementation and the illustrative example in the main text. Alternative normalizations would rescale the stochastic coordinate but would not affect the structural deviation nor the qualitative distinction between anchored and symmetric entropy lifts.

REFERENCES

1. Rencher, A.C., Schaalje, G.B.: *Linear Models in Statistics*. John Wiley & Sons, Hoboken, NJ (2007). 2nd ed.

2. Chow, G.C.: Tests of equality between sets of coefficients in two linear regressions. *Econometrica* **28**(3), 591–605 (1960)

3. Cover, T.M., Thomas, J.A.: *Elements of Information Theory*. John Wiley & Sons, Hoboken, NJ (2006). 2nd ed.

4. Shannon, C.E.: A mathematical theory of communication. *Bell Syst Tech J* **27**(3), 379–423 (1948). https://doi.org/10.1002/j.1538-7305.1948.tb01338.x
